# Prognostic impact of HLA-G expression in cervical squamous cell carcinoma: correlation with PD-L1 and CD8-positive tumor infiltrating lymphocytes

**DOI:** 10.3389/fimmu.2026.1957106

**Published:** 2026-09-11

**Authors:** Yi-Xiao Pan, Wei-Fei Chen, Rong Li, Meng-Jia Zhang, Zi-Yi Yan, Hui-Hui Xu

**Affiliations:** 1 Department of Public Research Platform, Taizhou Hospital of Zhejiang Province Affiliated to Wenzhou Medical University, Linhai, Zhejiang, China; 2 Department of Pathology, Taizhou Hospital of Zhejiang Province Affiliated to Wenzhou Medical University, Linhai, Zhejiang, China; 3 Medical Research Center, Taizhou Hospital of Zhejiang Province Affiliated to Wenzhou Medical University, Linhai, Zhejiang, China; 4 College of Laboratory Medicine and Life Sciences, Wenzhou Medical University, Wenzhou, Zhejiang, China; 5 Key Laboratory of Minimally Invasive Techniques & Rapid Rehabilitation of Digestive System Tumour of Zhejiang Province, Linhai, Zhejiang, China

**Keywords:** CD8, cervical squamous cell carcinoma, HLA-G, ILT2, immune checkpoint (ICIs), PD-L1, tumor-infiltrating lymphocytes

## Abstract

**Background:**

Purpose of the study was to investigate the prognostic impact of HLA-G expression in patients with cervical squamous cell carcinoma (CSCC), and to further analyze its correlations with PD-L1 and CD8-positive (CD8^+^) tumor-infiltrating lymphocytes (TILs) within the tumor microenvironment (TME).

**Methods:**

A total of 139 formalin-fixed paraffin-embedded (FFPE) tissue samples were collected from patients diagnosed with CSCC. Immunohistochemistry (IHC) and multiplex immunofluorescence (mIF) staining were performed to assess the expression of immune biomarkers, including HLA-G, its receptor ILT2, PD-L1, total TILs and CD8^+^ TILs.

**Results:**

The overall positive expression rates were 86.8% for HLA-G, 85.1% for sHLA-G, 50.0% for PD-L1, and 70.2% for cases with low TIL infiltration in the TME. These immune biomarkers displayed distinct immunoreactivity patterns with remarkable intratumoral and stromal heterogeneity. ILT2 expression was positively correlated with CD8^+^ TIL infiltration in both intratumoral and stromal compartments, yet rare cellular co-localization between ILT2 and CD8 was detected. Within tumoral regions, a strong positive correlation was identified between HLA-G and PD-L1 expression, and the two proteins were confirmed to be co-localized at the single-cell level in SCC lesions. Using the median HLA-G expression level of 61.0% as the cutoff value, patients with HLA-G expression above this threshold showed significantly worse overall survival (OS). The subgroup with high HLA-G/sHLA-G and low TIL infiltration showed the worst prognosis.

**Conclusions:**

The present study confirms that elevated HLA-G/sHLA-G expression serves as an independent adverse prognostic factor for cervical cancer. Combined assessment of HLA-G, PD-L1 and TIL infiltration may enable risk-stratified prognostic management for patients with cervical cancer.

## Background

Cervical cancer, the fourth most common malignancy in women worldwide, has become a major focus in immunotherapeutic research. The efficacy of such treatments is highly dependent on the tumor microenvironment (TME), which plays a critical role in regulating disease progression and therapeutic response of cervical cancer (1). In patients with PD-L1-positive recurrent or metastatic cervical cancer, anti-PD-1 monotherapies (such as pembrolizumab, nivolumab, or cemiplimab) have shown certain therapeutic advances; however, their objective response rate (ORR) remain suboptimal (14.3% with pembrolizumab and 26.3% with nivolumab) (2). This underscores the need to identify potential immune-related prognostic biomarkers. Recent studies have shown that human leucocyte antigen-G (HLA-G) expression in cervical lesions is associated with HPV infection and the host immune response (3, 4). Aberrant HLA-G expression in cervical lesions may be triggered by HPV infection. Subsequent upregulation of HLA-G further induces progressive impairment of T cell activity, which sustains persistent HPV infection and exacerbates immune tolerance in the lesion microenvironment (5). Furthermore, increased HLA-G levels are strongly related to higher tumor grades and worse prognosis in cancer patients (6–8). Due to its aberrant expression pattern and critical immunosuppressive roles in tumorigenesis, HLA-G has emerged as a novel and promising immune checkpoint target for cancer immunotherapy.

Unlike other HLA class I antigens, HLA-G undergoes alternative splicing of its primary transcript, generating seven mRNA variants that encode four membrane-bound (HLA-G1 to -G4) and three soluble (HLA-G5 to -G7) isoforms. HLA-G mediates immunosuppression by binding to their inhibitory receptors (e.g., ILT2, ILT4, KIR2DL4), thereby impair antitumor immune response. Specifically, HLA-G expressed on tumor cells interacts with its receptors ILT2 on T cells. This spatial distribution enables receptor-ligand complexes, which induces T cell exhaustion and suppresses the cytotoxicity of CD8^+^ T cells (CTLs), with both mechanisms enabling tumor immune escape (3, 9, 10). Evidence showed that HLA-G expression by target cells directly inhibits the cytotoxicity of CD8^+^ILT2^+^ T cells, highlighting the functional significance of this specific receptor-ligand axis in blunting antitumor immunity; concurrently, these findings underscore the therapeutic potential of targeting the HLA-G/ILT2 checkpoint in HLA-G^+^ tumors patients, who might profit from immunotherapeutic strategies (11). Despite its well-characterized immunoregulatory properties in promoting immune evasion across various malignancies, the prognostic relevance of HLA-G in cervical cancer—particularly when considered alongside the infiltrative patterns and functional status of CD8^+^ tumor-infiltrating lymphocytes (TILs)—remains poorly defined.

Therefore, this study aimed to explore the prognostic impact of HLA-G expression in CSCC patients, to further analyze its spatial correlations between HLA-G and PD-L1 as well as CD8^+^ TILs within the TME, and to determine the combined prognostic performance of these immune markers for patient clinical outcomes.

## Materials and methods

### Patient population

Between 2008 and 2022, a total of 139 patients with primary squamous cell carcinoma (SCC) who received surgical treatment at Taizhou Hospital were enrolled in this study. The cohort had a median age of 51.0 years (range: 33–82 years). No patients underwent chemotherapy or radiotherapy prior to surgery. The detailed clinicopathological characteristics of the study population were summarized in **Table 1**. All patients included in this retrospective cohort were retrospectively re-staged according to the FIGO 2018 cervical cancer staging system. The mean survival time of patients was 103.5 ± 68.2 months, with a range of 2 to 231 months.

**Table 1:** Clinicopathological characteristics of the study population.

Characteristics	n = 139 (%)
Age (range, 33-82 years)	
≤ Median (51 years)	70 (50.4)
> Median	69 (49.6)
FIGO Stage	
I	60(43.2)
II	67(48.2)
III	11(7.9)
IV	1(0.7)
Nodal status	
Negative	86(61.9)
Positive	33(23.7)
Unknown	20(14.4)
Follow-up	
Alive	107(77.0)
Death	30(21.6)
Lost	2(1.4)

### Immunohistochemistry staining

Serial 4-μm thick sections were prepared to detect the following immunomarker proteins in the TME: HLA-G (4H84, 1:100; Abcam), soluble HLA-G (sHLA-G) (5A6G7, 1:300; Abcam), ILT2 (EPR21007, 1:300; Abcam), PD-L1 (MAB1561, 1:400; R&D), and CD8 (C8/144B, 1:300; Abcam). mAb 4H84 is an IgG1 antibody specific for HLA-G, which recognizes the denatured HLA-G heavy chain of all HLA-G isoforms (HLA-G1 to -G7). mAb 5A6G7, also an IgG1 antibody, targets the denatured heavy chain of soluble HLA-G isoforms (HLA-G5 and HLA-G6). Briefly, the sections were first deparaffinized in xylene, rehydrated through graded ethanol, and blocked for endogenous peroxidase activity to prevent nonspecific staining. Following antigen retrieval, all sections were washed in TBS buffer and incubated with primary antibodies overnight at 4 °C. Antibodies were visualized using an HRP-conjugated secondary antibodies system (Dako, Glostrup, Denmark). Finally, the sections were counterstained with hematoxylin, dehydrated, and mounted for microscopic observation.

### Imaging and scoring

All IHC-stained sections were independently assessed and scored by two experienced pathologists (Pan YX and Yan ZY), both of whom were blinded to the patients’ clinical and pathological information. Based on the previously validated scoring protocol, tumor tissues with target immunomarker expression in more than 5% of tumor cells were defined as positive cases according to the tumor proportion score (TPS) (12, 13). Positive staining results were further stratified into four grades according to the positive cell proportion: 1+ (6-25%), 2+ (26-50%), 3+ (51-75%), and 4+ (>75%). Cells were categorized as immunopositive solely based on the presence of target biomarker staining, without accounting for staining intensity (**Supplementary Figure 1**).

### Multiplex immunofluorescence staining

mIF staining was conducted using the Opal 6-Color Manual IHC Kit (PerkinElmer, Waltham, MA, USA) in strict accordance with the manufacturer’s instructions. Briefly, 4-μm FFPE tissue sections were incubated with primary antibodies targeting HLA-G, ILT2, PD-L1, CD8, and pan-CK (1:1000; Abcam), followed by HRP-conjugated secondary antibodies combined with tyramide signal amplification (TSA). Cell nuclei were counterstained with DAPI for 10 minutes. Finally, stained tissue sections were digitally scanned and imaged using the TissueFAXS imaging system (TissueGnostics GmbH, Vienna, Austria). For each sample, manual regional annotation combined with automated cell counting was used to measure cell density in both intratumoral and stromal regions of invasive carcinoma tissues.

### Tumor infiltrating lymphocytes assessment

To quantify intratumoral TILs (tTILs) and stromal TILs (sTILs) within the TME, morphological assessment was performed in accordance with the guidelines of the International Immuno-Oncology Biomarker Working Group (14, 15). Briefly, pathologists first delineated “tumor” and “stromal” regions on H&E slides under low-power magnification, then scored the percentage of tTILs and sTILs for each sample. Patients were stratified into high- and low-infiltration subgroups using the median lymphocyte infiltration density as the cutoff value, with a threshold of 13.5% for total TILs (defined as the mixed pool of all lymphocytes in the TME). By contrast, mIF analysis quantified CD8^+^ T cells, a key cytotoxic subset within total TILs, and reported cell density as cells/mm². Separate median-derived cutoffs were applied: 166.1 cells/mm² for the intratumoral compartment and 499.4 cells/mm² for the stromal compartment.

### Statistical analysis

All statistical analyses were performed using SPSS version 21.0 and GraphPad Prism version 8.0. A two-tailed *P* value < 0.05 was considered statistically significant. Nonparametric intergroup comparisons were conducted via the Wilcoxon signed-rank test for two paired groups. The Pearson’s χ² test and Fisher’s exact test were adopted to analyze categorical and binary variables, respectively. Spearman’s rank correlation analysis was applied to assess correlations between the measured biomarkers. Survival curves were plotted using the Kaplan–Meier approach, and the log-rank test was used for intergroup survival comparisons. Multivariable Cox proportional hazards regression model, adjusted for age, FIGO stage, and lymph node status, was utilized to explore the associations between clinical covariates and patient survival. Overall survival (OS) was defined as the time from initial diagnosis to all-cause death or the last follow-up date (October 21, 2025).

## Results

### Expression of immunological markers

**Figure 1:**
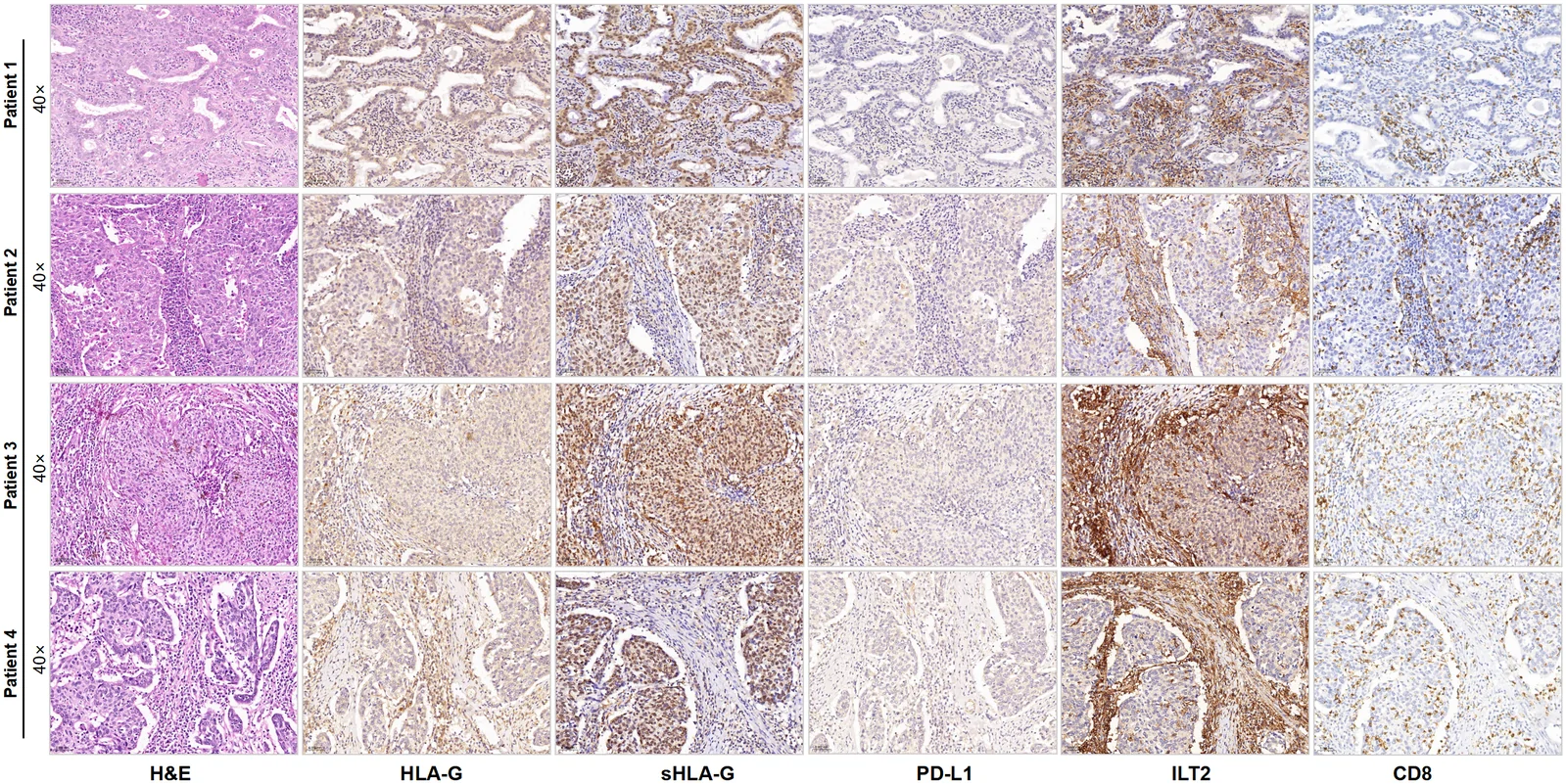
Representative IHC images showing the localization of immune biomarkers within the TME. The protein expression profiles of HLA-G, ILT2, PD-L1, and CD8 was evaluated in cervical cancer tissues, with corresponding hematoxylin and eosin (H&E) staining provided as a morphological reference. Scale bar is 50 μm (40×).

IHC staining for total HLA-G, sHLA-G, PD-L1, ILT2, and CD8 in cervical SCC tissues showed intense immunoreactivity with prominent intratumoral and stromal heterogeneity. The overall positive expression rates within the TME were 86.8% for HLA-G, 85.1% for sHLA-G, 50.0% for PD-L1, and 70.2% for cases with low TIL infiltration. Representative IHC images are presented in **Figure 1**, illustrating heterogeneous immunoreactivity of these immune biomarkers within the TME. sHLA-G expression was predominantly expressed in tumor cells relative to stromal cells, whereas ILT2 was predominantly localized to stromal cells rather than tumor cells. PD-L1 showed widespread positive staining in both tumor and stromal cells.

### Co-expression of immune checkpoint molecules

**Figure 2:**
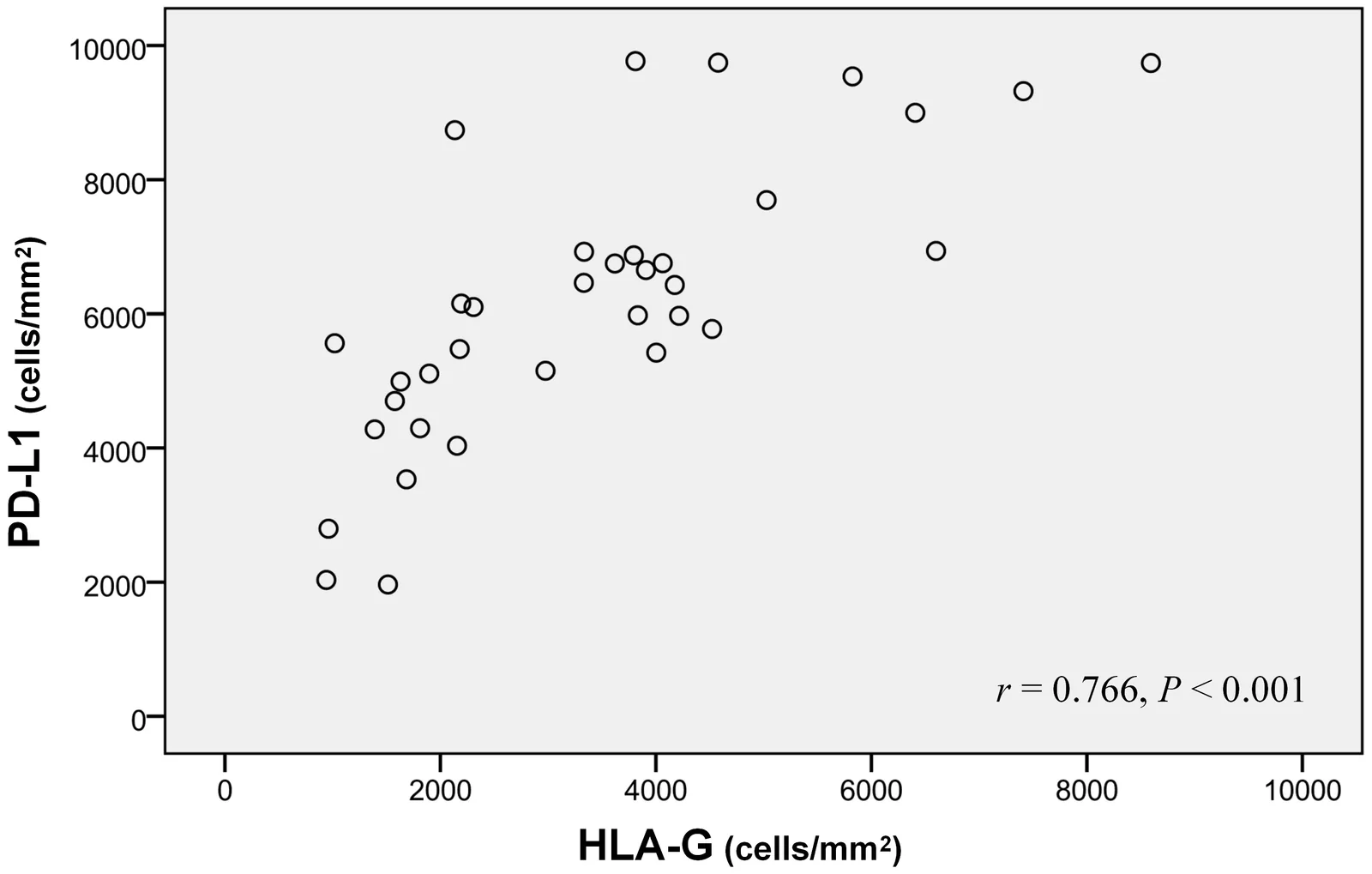
Spearman’s correlation analysis for the expression of HLA-G and PD-L1 within tumor tissues. HLA-G expression exhibited a strong positive correlation with PD-L1 expression (*r* = 0.766, *P* < 0.001).

**Figure 3:**
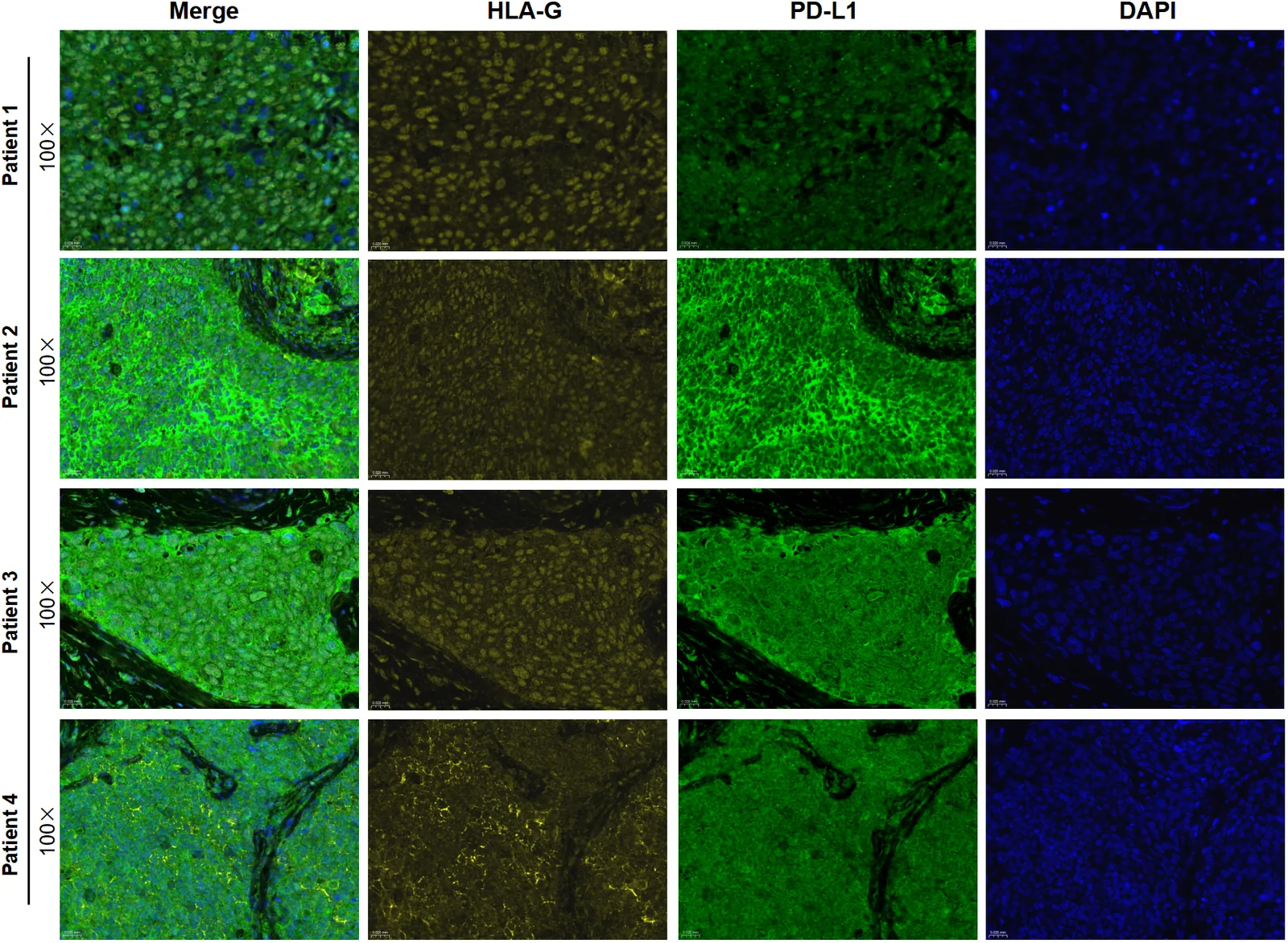
Representative mIF images showing the co-expression of HLA-G and PD-L1 within the TME. HLA-G and PD-L1 exhibited prominent co-expression. Scale bar is 20 μm (100×).

Within intratumoral regions, the median density of HLA-G^+^ cells was 3333.8 cells/mm^2^, whereas the median density of PD-L1^+^ cells reached 6102.4 cells/mm^2^. Spearman’s rank correlation analysis confirmed a strong positive correlation between HLA-G and PD-L1 expression in tumor tissues (*r* = 0.766, *P* < 0.001; **Figure 2**). Representative mIF images are shown in **Figure 3**, visualizing the colocalized co-expression of HLA-G and PD-L1 in the TME. Among HLA-G-positive tumor samples, the PD-L1 co-expression proportion varied from 63.6% to 100%.

### Correlations among CD8^+^ TILs and immunological markers

**Figure 4:**
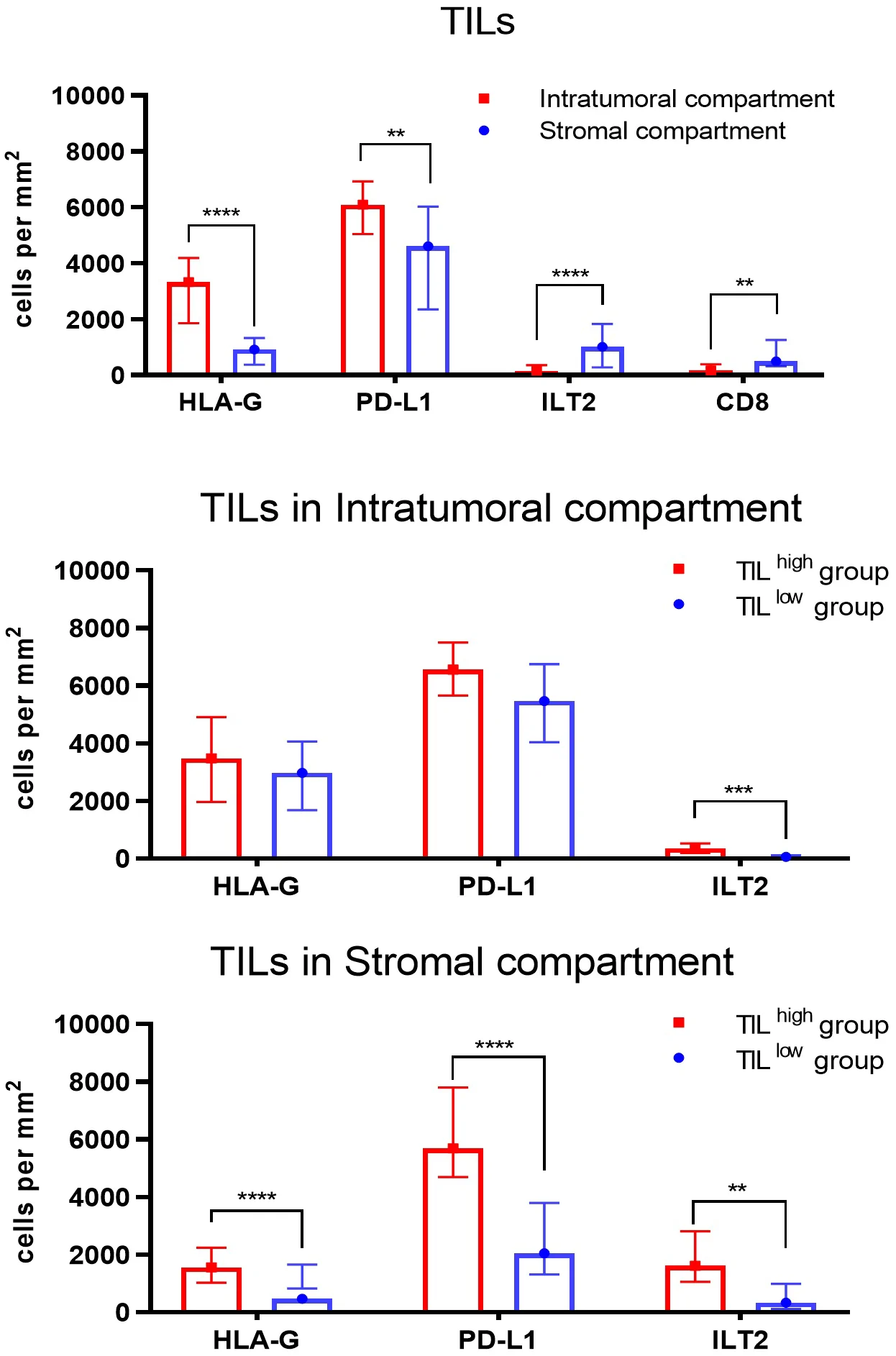
Quantitative differences in the expression levels of immune markers in tumor tissues stratified by high and low CD8^+^ TIL infiltration status. All data are expressed as median (25th-75th percentiles). *P < 0.05, **P < 0.01,***P < 0.001,****P < 0.0001.

**Figure 5:**
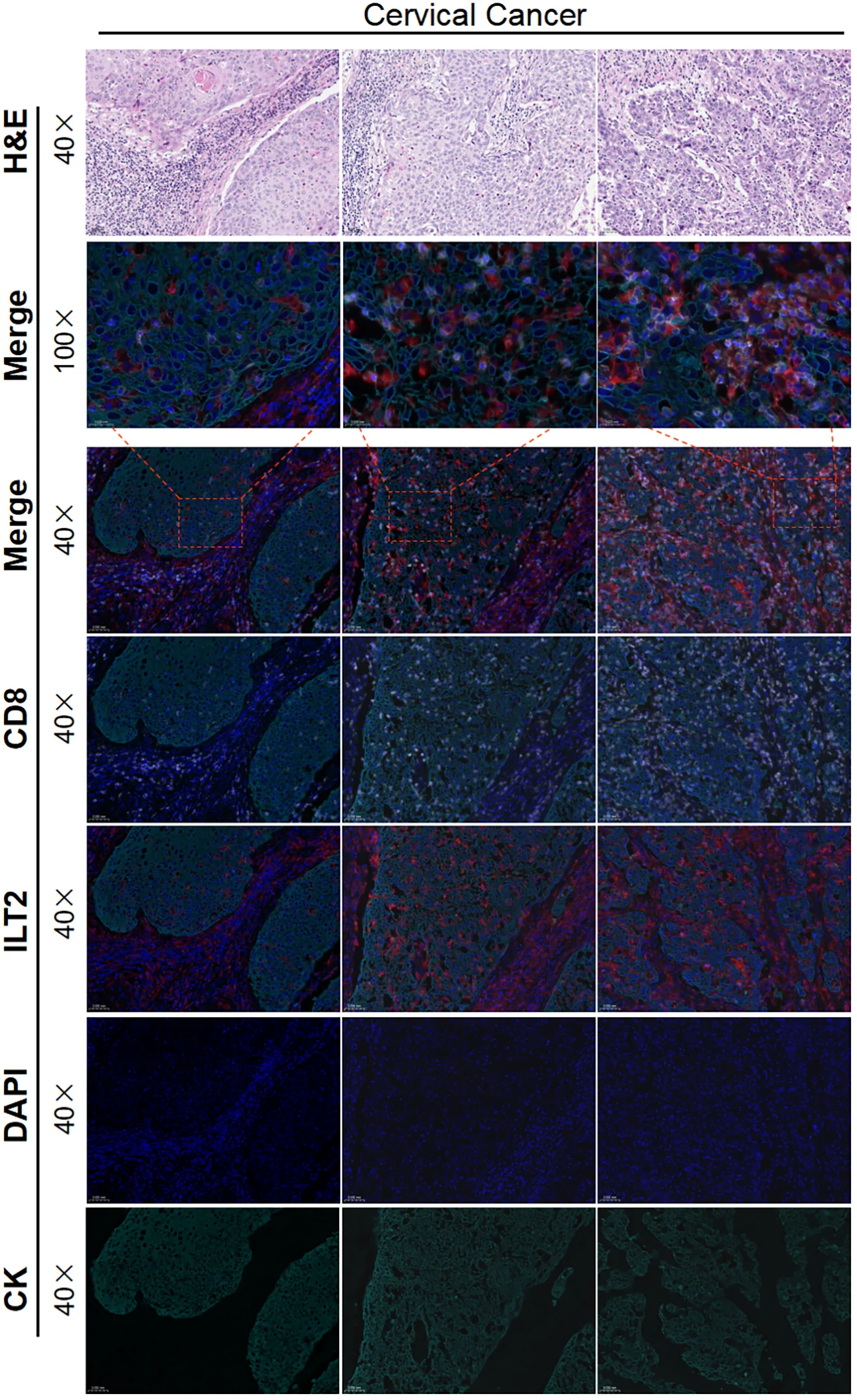
Representative mIF images showing the co-localization of ILT2 and CD8 within the TME. Cellular co-localization between ILT2 and CD8 was rarely observed in single cells. Scale bar is 20 μm (100×).

Median values and ranges of HLA-G^+^, PD-L1^+^, and ILT2^+^ cell densities (cells/mm^2^) in intratumoral and stromal compartments, stratified by low *vs.* high CD8^+^ TIL infiltration status are presented in **Figure 4**. Patients were dichotomized into two subgroups based on the median cutoff of CD8^+^ T cell density: 166.1 cells/mm^2^ for the intratumoral region and 499.4 cells/mm^2^ for the stromal region. Moreover, ILT2 density differed significantly between intratumoral and stromal compartments (*P* < 0.01). ILT2 expression levels were positively correlated with CD8^+^ TIL infiltration in both intratumoral (*r* = 0.828, *P* < 0.001) and stromal compartments (*r* = 0.796, *P* < 0.001). Nevertheless, cellular colocalization of ILT2 and CD8 was rarely detected in individual cells (**Figure 5**).

### Immunological markers and survival

**Figure 6:**
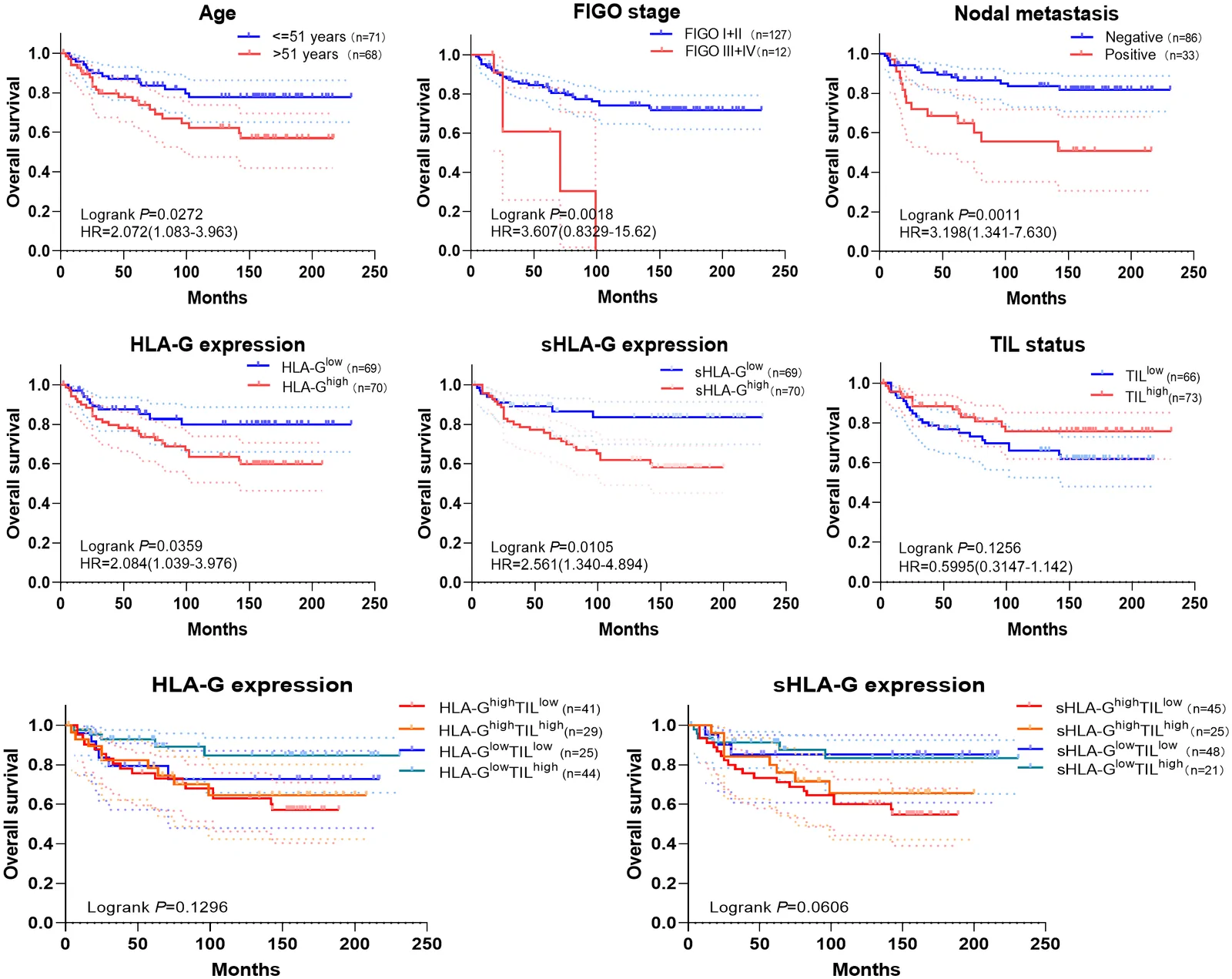
Kaplan–Meier curves for overall survival of patients with cervical cancer. The x-axis represents survival time, and the y-axis represents survival probability. Prognostic analysis was performed based on total TILs, representing the mixed pool of all lymphocytes in the TME.

Kaplan–Meier survival curves and Cox regression analyses are presented in **Figure 6** and **Table 2**. Univariate analysis revealed that the OS of patients with SCC was significantly associated with tumor FIGO stage (FIGO I+II *vs.* III+IV, hazard ratio [HR] = 3.61), lymph node metastasis (pN^-^ *vs.* pN^+^, HR = 3.20), and age at initial diagnosis (≤ 51 years *vs.* > 51 years, HR = 2.07). The median expression level of each biomarker was used as the cutoff value to stratify patients into high- and low-expression subgroups: 61.0% for total HLA-G, 71.0% for soluble HLA-G, and 13.5% for total TILs. Patients with high expression of total HLA-G or soluble HLA-G exhibited significantly worse OS (all *P* < 0.05). By contrast, patients with high TIL infiltration had longer OS, although this difference did not reach statistical significance. Furthermore, a multivariate Cox regression analysis revealed that total HLA-G and soluble HLA-G may serve as independent poor prognostic factor for cervical cancer (all *P* < 0.05) (**Table 2**).

**Table 2:** Univariate and multivariate analysis on the impact of prognostic factors for overall survival.

Categories	Kaplan-Meier analysis		Cox regression analysis	
	HR (95% CI)	*P*-value	HR (95% CI)	*P*-value
Age (≤ 51 years *vs.* > 51 years)	2.07(1.08-3.96)	0.03	2.65(1.01-6.94)	0.048
Stage (FIGO I+II *vs.* III+IV)	3.61(0.83-15.62)	0.002	–	–
Lymph-node status (pN^-^ *vs.* pN^+^)	3.20(1.34-7.63)	0.001	1.46(0.62-3.42)	0.001
HLA-G^+^ (low *vs.* high)	2.08(1.04-3.98)	0.04	3.26(1.32-8.05)	0.011
sHLA-G^+^ (low *vs.* high)	2.56(1.34-4.89)	0.01	3.66(1.37-9.79)	0.010
TIL status (low *vs.* high)	0.60(0.25-1.43)	0.24	0.69(0.28-1.71)	0.420

HR, hazard ratio; 95% CI, 95% confidence interval. Patients were stratified into high- and low-expression subgroups using the median value of each biomarker as the cutoff threshold: 61.0% for HLA-G, 71.0% for sHLA-G, and 13.5% for total TILs. Multivariate Cox regression analysis was performed with adjustment for age and lymph-node status.

### Combined prognostic impact of HLA-G and TILs

As depicted in **Figure 6**, patients with high HLA-G expression combined with low TIL infiltration had the worst prognosis. Patients with high HLA-G/high-TIL and low HLA-G/low-TIL status exhibited progressively better-outcomes in that order. The same trend was also observed in the sHLA-G subgroup. Collectively, these findings suggest that a combined prognostic effect of HLA-G and TILs in cervical cancer patients.

## Discussion

Immune checkpoint blockade (ICB) mediated by therapeutic monoclonal antibodies has revolutionized the treatment landscape of cervical cancer through interruption of oncogenic ligand-receptor crosstalk (16). To date, anti-PD-1 monotherapy has demonstrated notable efficacy in recurrent/metastatic cervical cancer; however, the majority of PD-L1-positive patients fail to achieve durable clinical responses, suggesting that multiple non-redundant immunosuppressive pathways may coexist and exert synergistic effects within the TME (2, 17). HLA-G is an emerging tumor biomarker that actively regulates key malignant phenotypes, including tumor initiation, cell proliferation, invasion, and metastasis (3, 9). Given its restricted expression in healthy tissues and potent immunoinhibitory functions, HLA-G has been recognized as a novel immune checkpoint, which renders it an attractive candidate target for ICB therapeutic strategies (10). Cytotoxic CD8^+^ T cells represent the primary effector population responsible for anti-tumor immune surveillance. The functional states and abundances of intratumoral CD8^+^ TILs in TME across different cancer types fundamentally influence clinical outcomes, including drug response to immunotherapy (18). In this context, comprehensively dissecting the complex crosstalk among HLA-G (both membrane-bound and soluble isoforms), PD-L1, and CD8^+^ TILs within the TME is critically important. Elucidation of such immune regulatory networks may help resolve the current therapeutic limitations of conventional PD-1/PD-L1 blockade and facilitate the development of more precise, effective, and personalized immunotherapeutic regimens for cervical cancer.

In this study, we identified increased expression of the immune checkpoint HLA-G in tumor tissues from patients with SCC. Clinically, overexpression of HLA-G was strongly associated with unfavorable prognostic outcomes, and soluble HLA-G isoforms displayed comparable prognostic implications for SCC patients. Moreover, we further verified HLA-G expression was positively correlated with PD-L1 expression, and the two proteins were co-localized at the cellular level in SCC lesions. Our findings support recent studies showed high HLA-G expression alongside PD-L1 in colorectal cancer and clear cell renal cell carcinoma (ccRCC) (19, 20). Existing mechanistic studies have confirmed that cellular differentiation or chemotherapeutic stress can markedly upregulate HLA-G and PD-L1 expression, thereby promoting tumor immune escape and ultimately inducing the development of chemotherapy and immunotherapy resistance (21, 22). Therapeutically, this crosstalk between HLA-G and PD-L1 provides a plausible mechanistic explanation for the immune escape of certain tumors following anti-PD-1/PD-L1 immunotherapy.

Furthermore, our study revealed significant differences in survival outcomes between patients with high *vs.* low expression of total or soluble HLA-G isoforms, and HLA-G expression could serve as an independent poor prognostic factor for cervical cancer. Survival stratification analysis showed that patients with HLA-G^high^TIL^low^ expression had the worst overall survival, followed by those with HLA-G^high^TIL^high^ and HLA-G^low^TIL^low^ expression. These findings provide clinical evidence that HLA-G, as an immune checkpoint, may modulate the prognostic impact of total TILs in cervical cancer. This observation aligns well with its established biological role in mediating tumor immune escape by inhibiting the cytotoxic activity of T cells within the TME (23, 24). In line with the above findings, our data further verified that ILT2, a receptor for HLA-G, was positively correlated with CD8^+^ TIL infiltration. Nevertheless, it is noteworthy that cellular colocalization of ILT2 and CD8 was rarely detected at the single cell level within the TME. This implies that immune cells involved in HLA-G-ILT2 crosstalk are not limited to CD8^+^ T lymphocytes. Previous study has reported that HLA-G interacts with ILT2 expressed on CD4^+^ T cells in the ccRCC microenvironment (12), thereby facilitating tumor-cell evasion of anti-tumor immunity.

Collectively, the present findings validate prior evidence that HLA-G would generate immune resistance of T cell inactivation in the TME, and that HLA-G overexpression is associated with advanced disease stages and poorer clinical outcomes among patients with cervical cancer. Consistent with our results, increased circulating HLA-G and its receptor ILT2 have been closely associated with therapeutic resistance (25, 26). Moreover, HLA-G^high^ PD-1^-^ILT2^+^ cytotoxic T cells in renal cancer exhibit selective functional suppression, and this inhibitory effect can only be reversed by HLA-G blockade (27). Therefore, targeted blockade of the HLA-G/ILT2 signaling pathway may confer prolonged therapeutic efficacy with minimal systemic toxicity (28–30). Notably, combined blockade of PD-L1 and HLA-G may represent a promising and novel therapeutic strategy.

This study has several notable limitations. Although we performed harmonized retrospective restaging according to the FIGO 2018 criteria and adjusted for major clinical and therapeutic confounders in the Cox regression model, unmeasured confounders cannot be fully excluded. We could not further delineate the precise subsets of ILT2-positive tumor-infiltrating cells in the cervical tumor microenvironment, and the immune functions mediated by HLA-G-ILT2 interactions remains incompletely defined. Future single-cell-based studies are warranted to resolve this issue. Furthermore, the present study did not explore the functional status of CD8^+^ TILs in the cervical cancer tumor microenvironment. Accumulating evidence has demonstrated the functional impacts and clinical relevance of exhausted CD8^+^ T cells across various solid-tumor types (31, 32). Future functional investigations are necessary to further characterize CD8^+^ T cells subsets in the TME.

In conclusion, the present study confirms that high HLA-G expression serves as an independent poor prognostic factor for cervical cancer. HLA-G can serve as a promising prognostic biomarker and potential therapeutic target. Combined assessment of HLA-G, PD-L1 and CD8^+^ TIL enables prognostic risk-stratified management in patients with cervical cancer.

## Data Availability

The original contributions presented in the study are included in the article/**Supplementary Material**. Further inquiries can be directed to the corresponding author.

## Supplementary material

Supplementary Figure 1 Representative IHC images of HLA-G negative and positive expression cases. sHLA-G protein was detected using mAb 5A6G7. Scale bar is 20 μm (100×), 100μm (20×).
